# Differentially private distributed logistic regression using private and public data

**DOI:** 10.1186/1755-8794-7-S1-S14

**Published:** 2014-05-08

**Authors:** Zhanglong Ji, Xiaoqian Jiang, Shuang Wang, Li Xiong, Lucila Ohno-Machado

**Affiliations:** 1 Division of Biomedical Informatics, University of California, San Diego, CA 92093, USA; 2 Department of Mathematics and Computer Science, Emory University, Atlanta, GA 30322, USA

## Abstract

**Background:**

Privacy protecting is an important issue in medical informatics and differential privacy is a state-of-the-art framework for data privacy research. Differential privacy offers provable privacy against attackers who have auxiliary information, and can be applied to data mining models (for example, logistic regression). However, differentially private methods sometimes introduce too much noise and make outputs less useful. Given available public data in medical research (e.g. from patients who sign open-consent agreements), we can design algorithms that use both public and private data sets to decrease the amount of noise that is introduced.

**Methodology:**

In this paper, we modify the update step in Newton-Raphson method to propose a differentially private *distributed *logistic regression model based on both public and private data.

**Experiments and results:**

We try our algorithm on three different data sets, and show its advantage over: (1) a logistic regression model based solely on public data, and (2) a differentially private distributed logistic regression model based on private data under various scenarios.

**Conclusion:**

Logistic regression models built with our new algorithm based on both private and public datasets demonstrate better utility than models that trained on private or public datasets alone without sacrificing the rigorous privacy guarantee.

## Introduction

Data about individuals are being collected at an unprecedented speed, which brings new opportunities for scientific discovery and healthcare quality improvement. In the meantime, there is increasing concern about people's privacy and inappropriate disclosure of sensitive information [1]. This problem is especially challenging in biomedicine [2], where information sharing is one of the biggest pillars to facilitate meaningful analysis of complex medical data. For example, classifying complex or rare patterns in clinical and genomic data requires the availability of a large, labeled patient set, which needs to be obtained from multiple institutions [3].

Any data access mechanism involves a tradeoff between the privacy risk and the data utility. In biomedicine, data custodians can change the content of the data to make more difficult for attackers to re-identify individuals (k-anonymity [4], l-divergence [5], t-closeness [6], etc.) or can perturb the outputs of a query result to ensure "indistinguishability" of individuals (i.e., count queries satisfying differential privacy [7]). Because *differential privacy *[8] provides a provable guarantee and is immune to attacks with auxiliary information, it is acknowledged as a state-of-the-art privacy definition [9]. The performance (i.e., privacy and utility) of a differentially private method is highly dependent on the nature of the application and the capability of the protection mechanism. To meet the need of different applications, many customized differentially private methods, including decision trees [10], logistic regression [11], principal components analysis [12], multi-class Gaussian classifiers [13], have been developed. There are several recent efforts in integrating the differential privacy framework into the system design and case studies for statistical health information release [14], [15]. However, due to an inherited challenge of differential privacy in considering the entire sample space, the level of data perturbation often increases too quickly when the privacy assurance becomes stronger, which ends up adding too much noise [16], i.e., producing useless, albeit protected, data.

We believe the situation can be alleviated in an environment where both public and private data sets for the same study are available for analysis. This is useful in biomedical research (e.g., randomized clinical trial), where some patients are willing to sign an open-consent agreement to make their data (publicly) available for research, while other patients prefer to limit disclosure to a single institution. Our idea is to develop hybrid data mining models using both public and private data sets in a differentially private and distributed manner to achieve improved utility of the disclosed data. We will focus on the logistic regression model, which is one of the most popular approaches in biomedicine, to develop a distributed and privacy preserving solution in the healthcare context.

### Related work

Our model is closely related to Grid Logistic Regression, a model developed by Wu [3]. Their model is based on a distributed Newton-Raphson algorithm, however, it does not consider privacy risk during the exchange of aggregated statistics among participants. A recent work by Wu et al. [17] discusses institutional privacy of distributed logistic regression and introduces a secure-sum based approach to protect aggregated statistics using a trusted server, but it does not meet the differential privacy criteria. The underlying intuition of our model is close to Elkan's work [18] to represent a confidential database via importance weighting elements of a public database for general data mining purpose, but his approach is also not differentially private.

There are also some previous works on differentially private distributed learning. For example, Pathak, Rane, Raj et al. [19] suggested running local models on each private data set and aggregating estimated parameters. This approach is different from ours in two aspects. First, their model does not take public data into consideration. Second, they only prove that the final outputs (the aggregated parameters) are differentially private, while there is no guarantee that the intermediary outputs from individual private data sets (which need to be shared during the process) are differentially private. In contrast, our model ensures differential privacy for all steps. Rajkumar and Agarwal [20] recently proposed a distributed differentially private stochastic gradient descent algorithm, which also differs from ours as follows: (1) their approach used only private data; (2) their approach is (ϵ,δ)-differentially private (weaker) while ours is -differentially private (stronger).

In this paper, we introduce a new distributed logistic regression model that runs on many data sets, e.g., both public and private ones. It treats these two kinds of data sets differently: it leverages public data sets to improve utility while protecting the private data sets. Background section introduces some background knowledge and methodology section elaborates on details of our method. In experiments and results section, we compare our model to other approaches, and explore the impact of different settings (i.e., the fraction of data that are public, the number of distributed private data sets, and impact of the regularization parameter) on the final model. Finally, discussions and conclusions are drawn.

## Background

In this Section, we will briefly review techniques related to this article.

### Differential privacy

Differential privacy (DP) is a privacy definition proposed by Dwork, Kenthapadi, McSherry et al [8], which states that any answer to a *query *based on a private data set should not be altered dramatically with the change of a single record in the data set.

In the following part of this paper,  D and $D^{\prime }$ always differ on at most one sample (i.e.,  D and $D^{\prime }$ are "neighbors" with only one sample replaced).

**Definition 1: Query function **Function $f:D\to R^{p}$ is a query function, if it is a projection from a data set  D (not a single sample, but the whole data set) to $R^{p}$.

Many data mining models can be viewed as a query function, for example, the coefficients of the *logistic regression *can be seen as the projection of a data set to a real-valued vector.

**Definition 2: ****-Differential Privacy **A randomized algorithm (or mechanism) $\tilde{f}$ is -differentially private if for any neighbors  and  and for any $S\epsilon R^{p}$ when the following probabilities are well-defined,

$\text{Pr}\left[\tilde{f}\left(D\right)\epsilon S\right]\leq e^{\epsilon }\times \text{Pr}\left[\tilde{f}\left(D^{\prime }\right)\epsilon S\right]$,

where the probabilities reflect the randomness of the algorithm $\tilde{f}$. Note the parameter  is called **privacy budget**. The smaller  is, the better privacy is preserved, and vice versa.

**Definition 3: Sensitivity **A query function  f's sensitivity under norm ||.|| is defined by

$$s_{f,∥.∥}=\underset{D,D^{\prime }}{max}∥f\left(D\right)-f(D^{\prime })∥.$$

**Definition 4: Laplacian mechanism **[21] For any query function $f:D\to R^{p}$, the algorithm returns $\tilde{f}\left(D\right)=f\left(D\right)+\delta$, where $\delta :p\left(\delta \right)\propto \text{exp}\left(-\frac{\delta \epsilon }{s_{f,||.||}}\right)$, satisfies differential privacy.

If two independent mechanisms are $\epsilon_{1}$ and $\epsilon_{2}$ differentially private, running them iteratively on the same data set will consume a privacy budget $\epsilon_{1}+\epsilon_{2}$, which is known as the *sequential composition property *of differential privacy [22].

Typically, $L_{1}$ norm is used in calculating sensitivity and applying Laplacian mechanism, in which case the noise on different dimensions are independent. In this paper, we consider the $L_{2}$ norm instead, such that the sensitivity of penalized logistic regression parameter (i.e., the outputs of a query function) can be bounded, as proved in Corollary 2 by Chaudhuri et al [11]. The $L_{2}$ norm has been used in previous differential private algorithms, e.g., the work of Chaudhuri [11] and Rajkumar [20].

### Newton method for logistic regression

The Newton method (also known as the Newton-Raphson method [23]) is an iterative approach that uses gradient to find roots of a real-valued differentiable function. Since a function's extrema are also the roots of its gradient, the Newton method can also be used to find twice differentiable function's extrema. Due to its efficiency in handling convex functions, (i.e., usually only a few iterations (five or six) are needed to reach a very high precision [24]), the Newton method is a popular numerical approach for building a logistic regression model [25]. Given the log-likelihood function of a logistic regression model L(β), the Newton method approaches the maximum likelihood coefficients estimate *β *with the following steps,

Initialize $\beta_{0}=0$,

Compute the gradient and Hessian matrix of L(β),

$$grad={\left(\frac{\partial L\left(\beta \right)}{\partial \beta }\right|}_{\beta =\beta_{0}}$$

$$H={\left(\frac{\partial^{2}L\left(\beta \right)}{{\partial \beta }^{2}}\right|}_{\beta =\beta_{0}}$$

Update $\beta_{0}=\beta_{0}-H^{-1}grad$ and repeat the second step until $\beta_{0}$ converges.

## Methodology

Our goal is to develop a distributed logistic regression model that effectively synthesizes data (public and private) across different sites (institutions) in a differentially private manner.

### Assumptions about the data set

To develop the model in the biomedical context, we will make some assumptions about the data sets. First, the number of samples in each data set is not very large. This assumption is reasonable as otherwise one data set is enough to build the model and many data sets will not bring much benefit. Second, the size of the public data set is significantly smaller than that of the private data sets. The reason is that by default biomedical data should be kept secure and private unless patients are willing to sign open-consent agreements to make their medical data available for research, which only applies to a small percentage of the total data. Third, we assume samples in different data sets follow the same predictive rule, which means $P\left(label\text{|}predictors\right)$ must be the same across all data sets. This is necessary for constructing a distributed logistic regression model that can provide useful information in biomedical research. In practice, such assumption can be verified by checking the goodness-of-fit (e.g., Hosmer-Lemeshow test [26]) of the fitted local and global models without sacrificing individual privacy. Last, we assume that the distribution of samples in different data sets (P(predictors)) are similar, although this assumption will be relaxed, as we will elaborate in the discussion section.

#### Notation

All these samples are Independent and Identically Distributed (i.i.d.), and each sample has a binary label yϵ{1,-1} and a predictor vector  x. The  k private data sets (namely $D_{1},...D_{k}$) have $n_{1},...,n_{k}$ samples respectively, and the  i-th sample in the  j-th data set is denoted as $\left(x_{i}^{j},y_{i}^{j}\right)$. The public data set $D_{0}$ has $n_{0}$ samples $\left(x_{i}^{0},y_{i}^{0}\right),i=1,\ldots ,n_{0}$. The penalized logistic regression maximizes the following log-likelihood function

$$L(\beta )=\overset{k}{\underset{j=0}{{\sum^{\text{​}}}^{\text{​}}}}\overset{n_{j}}{\underset{i=1}{{\sum^{\text{​}}}^{\text{​}}}}log\frac{1}{1+exp(-y_{i}^{j}\beta^{T}x_{i}^{j})}-\frac{\lambda }{2}{∥\beta ∥}^{2}$$

and the *β *that maximizes this log-likelihood function is the estimated parameter for the model.

Note that for the bounded sensitivity, if an intercept is introduced into the predictors, the corresponding parameter should be also regularized.

### Method description

Because the logistic regression model has no analytic solutions, we need to solve it using numerical methods like the Newton algorithm (see the Background section), which involves several iterations of optimization. As the distributed logistic regression model is supposed to be trained on multiple data sets, we need to implement a privacy-preserving information exchange mechanism to transmit intermediary results across private data sets. In addition, the *privacy budget *is limited and it must be split across iterations. To maintain data utility, we must balance the number of iterations and the privacy budget spent on each iteration in the training process.

To use the Newton-Raphson algorithm, we need to compute the gradient and the Hessian matrix from the data sets. The simplest way to ensure differential privacy is to add Laplacian noise to the gradient and the Hessian matrix, and use the noisy version of these intermediary results to update parameters. Theoretically, the impacts of additive noise in Laplacian mechanism (see Definition 4) tend to be much smaller when the number of samples approaches infinity, as the sensitivity of the gradient and the Hessian matrix is irrelevant to the size of a data set. In reality, however, the effects of noise on the gradient and the Hessian matrix (for parameter estimation) are quite different. For example, the gradient is usually affected by the additive noise than is the Hessian matrix. This is because the gradient has linear impact on the parameter updates. However, this is not the case for the Hessian matrix. Since the inverse of the Hessian matrix is used in the update step, even a little noise in the Hessian matrix can lead to large changes on the parameters being updated. Such change can become very large when the noise destroys the Hessian matrix's positive definiteness, which implies that a global optimal solution (like the one for the log-likelihood function of a traditional logistic regression model) may not be attained. Although we can threshold the eigenvalues of the Hessian matrix to ensure positive definiteness, this method might generate useless coefficients. Therefore, *the key to build a useful differentially private distributed logistic regression using the Newton-Ralphson algorithm is to reduce the noise in the Hessian matrix, especially reduce the chance of a non-positive definite Hessian matrix*.

Our approach is to leverage public data sets (i.e., contributed by patients who signed the open-consent agreement). In our hybrid framework, the Hessian matrix is estimated solely using the public data set, and we use public and private data sets to compute the gradient. To estimate the Hessian matrix using only public data, we leverage the following advantages: (1) The Hessian matrix from the public data set is positive definite. Therefore, the worst case discussed above is avoided even though the absolute sample error on the Hessian matrix might be larger than the noisy Hessian matrix from individual data sets. (2) The sensitivity of Hessian matrix is $O\left(p^{2}\right)$ when there are *p *features in each sample, while the sensitivity of the gradient is only O(p). Therefore, if we use the public data to calculate the Hessian matrix, the total sensitivity can be reduced from $O\left(p^{2}\right)$ to O(p), which increases the accuracy of update steps.

Unlike the traditional Newton-Raphson algorithm, which iterates until convergence, our method uses a fixed number of iterations for the following reasons. First, the original Newton method stops after parameters converge but our algorithm will never terminate due to the noise added in each step. Second, the stop decision in our case cannot be accurately determined by comparing the likelihood associated with $\beta_{new}$ and $\beta_{old}$ because it is possible that the noisy $\beta_{new}$ performs worse than $\beta_{old}$, which will never happen in the standard maximum likelihood estimation. Finally, a fixed number of iterations allows us to allocate privacy budgets easily, e.g., evenly split across iterations as in this work.

The details of our update step are illustrated in Algorithm 1, and the full model is described in Algorithm 2.

There are two some modifications that can further improve the performance. Please refer to Additional file 1, for these modifications and for the proof of differential privacy.

## Algorithm 1

Modified update step in distributed logistic regression

### Input

Private dataset $D_{1},...,D_{k}$, public dataset $D_{0}$, privacy budget for this iteration $\epsilon_{0}$, coefficient of penalty  λ, the upper bound of $L_{2}$ norm of samples  M, and old parameter $\beta_{old}$ obtained from the previous iteration.

### Output

Logistic regression parameter $\beta_{new}$.

1: Compute the Hessian matrix  H using only the public data set ( I is the identity matrix)

$$H=\overset{n_{0}}{\underset{i=1}{\sum }}\text{log}\frac{\text{exp}\left(-\beta_{old}^{T}x_{i}^{0}\right)}{{\left(1+\text{exp}\left(-\beta_{old}^{T}x_{i}^{0}\right)\right)}^{2}}-\frac{n_{0}\lambda }{\sum_{j=0}^{k}n_{j}}I$$

The coefficient $\frac{n_{0}\lambda }{\sum_{j=0}^{k}n_{j}}$ is an adjustment, as the number of samples to obtain  H here is different from number of samples to get gradients below.

2: Compute the gradient grad for each data set.

$$grad^{j}=noise^{j}+\overset{n_{j}}{\underset{i=1}{\sum }}\frac{y_{i}^{j}x_{i}^{j}}{1+\text{exp}\left(y_{i}^{j}\beta_{old}^{T}x_{i}^{j}\right)}$$

$$grad^{0}=\overset{n_{j}}{\underset{i=1}{\sum }}\frac{y_{i}^{0}x_{i}^{0}}{1+\text{exp}\left(y_{i}^{0}\beta_{old}^{T}x_{i}^{0}\right)}$$

where $noise^{j},j=1,\ldots ,k$ are iid vectors with density $p\left(noise^{j}\right)\propto exp\left(-\frac{\epsilon_{0}{∥noise^{j}∥}_{2}}{2M}\right)$

3: Aggregate all the gradients

$$grad=\overset{k}{\underset{j=0}{\sum }}grad^{j}-\lambda \beta_{old}$$

4: Output $\beta_{new}=\beta_{old}-\frac{n_{0}}{\sum_{j=0}^{k}n_{j}}H^{-1}grad$

## Algorithm 2

Distributed logistic regression

### Input

Private dataset $D_{1},...,D_{k}$, public dataset $D_{0}$, privacy budget for this iteration , coefficient of penalty  λ, the upper bound of $L_{2}$ norm of samples  M, and iteration times  l.

### Output

Logistic regression parameter  β.

1: $\epsilon_{0}=\epsilon /l$

2: Initialize logistic regression parameter β=0, a vector with the same length as  x.

3: Given the data sets, $\epsilon_{0},\lambda$ and  M, use  β as $\beta_{old}$, update  β with $\beta_{new}$ in Algorithm 1. Repeat for  l times

4: Output *β*

## Experiments and results

We will compare our algorithm with two baselines. The first is the *meta analysis *method. It first adds noise from distribution $p\left(noise^{j}\right)\propto exp\left(-\frac{\epsilon \lambda {∥{noise}^{j}∥}_{2}}{2M}\right)$ to logistic regression parameters learned from a private data set, where  M is the upper bound of predictors' $L_{2}$ norm. Then, it outputs weighted average (by number of samples in each data set) of these noisy parameters learned from  k private data sets. By weighting locally learned differentially private parameters, this method is similar to the method in Pathak, Rane, Raj et al[19]. The only difference is that we make outputs from each private data be shared differentially privately (rather than transmitting encrypted partial local outputs). Our second baseline is to train a logistic regression model with only public data and neglect the private data sets. Our evaluation metric is model discrimination, i.e., the Area Under the ROC curve (AUC). We will explore the performance of all three models using various parameter values. Unless explicitly illustrated in the Figures, default values for the following parameters are set as follows: number of private data sets (3), fraction of public data in the training set (2%), privacy budget  ϵ (1), number of iterations (2). The regularization strength  λ for all three models is selected from a range [10^−2^,10^6^] to maximize the expected AUC based on a 10-fold cross validation.

We used clinical data to conduct experiments, where the public and private data sets are split randomly. Each private data set contains roughly the same number of observations. We used a 60%/40% split for training and testing in all experiments.

### Data sets

We used three data sets, i.e., Schumacher's breast cancer [27], hospital discharge [28], and the SEER breast cancer [29]. A summary is showed in Table 1, which includes data description, number of attributes, number of samples, and the class label distribution. Table II lists the attribute description for each data set, where numerical attributes are indicated by "∗", and non-binary categorical attributes were converted into binary ones through dummy coding. For example, a categorical attribute of *c *categories will be converted into *c *− 1 binary covariates in dummy coding (e.g., 0 → (1,0),1 → (0,1) and 2 → (0,0) for the case *c *= 3). The class label attributes are shown in the last row of Table 2.

**Table 1:** Summary of data sets used in our experiments

Data set	Data set description	# of attributes	# of samples	Class distribution (negative/positive)
1	German breast cancer	9	686	43.6% / 56.4%
2	Hospital discharge	17	8,668	4.4% / 95.6%
3	SEER breast Cancer	37	55,000	21.0% / 79.0%

**Table 2:** Attribute description for each data set, where numerical attributes are indicated with "∗", non-binary categorical attributes were converted into binary representations through dummy coding and classification labels are shown in the last row.

Data set 1	Data set 2	Data set 3
**Hormonal therapy** 1. Yes, 2. No.	**Specimen** 1. Blood, 2. Urine, 3. sputum, 4. CSF	**Race **(25 categories)

Age*	Specific days*	Age*

**Menopausal status** 1.Premenopausal, 2. Postmenopausal	**Day of the week for collection** 1. Weekday, 2. Weekend	**Marital status **(6 categories)

Tumor size*	Age*	Histology*

Tumor grade* (Levels I, II, III)	**Day of the week for the final result** 1. Weekday, 2. Weekend	Number of nodes examined*
Number of positive nodes*	**Gender** 1. Male, 2. Female	Number of positive nodes*
Recurrence free Survival time* (in days)	**Insurance** 1.Medicare, 2. Medicaid, 3. Commercial, 4. Other	Grade*
Progesterone receptor*		Tumor size*
Estrogen receptor*	**Race** 1. White, 2. Black, 3. Asian, 4. Hispanic, 5. unknown/declined	**ER status** (4 categories)
**Status indicator**	**Potential error**	**Vital status recode**
Pos: Alive, Neg: Died	Pos: Not a potential follow-up error, Neg: A potential follow-up error	Pos: Alive, Neg: Died

As the magnitudes of attributes have large impact on the overall sensitivity of logistic regression parameters and the gradients of the log-likelihood function, we normalized all attributes and truncated their values to [−2,2] in order to bound the impact. Note that we used the mean and the variance of public data to conduct normalization, which does not incur a privacy cost.

## Results

We first used the hospital discharge data set to explore the effect of different parameters on model discrimination. Then, we evaluated and compared the model performance using all three datasets to check the impact of scalability.

**Figure 1:**
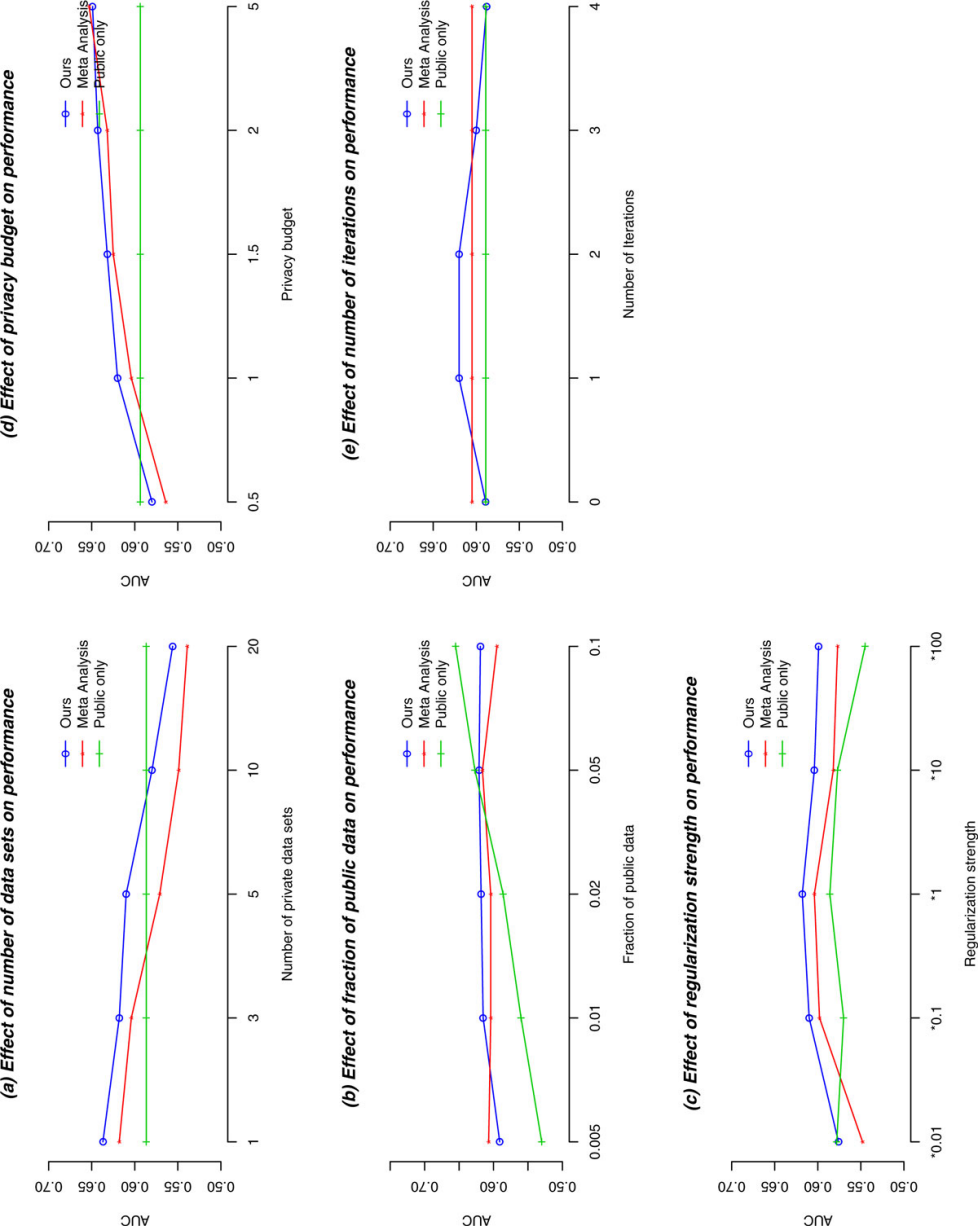
Effect of different parameters on model discrimination. Note that in 1(e), only our model is affected by iteration numbers.

*1) Model comparison using different parameters: *In Figure 1a, we illustrated the effect of different number of private data sets on discrimination. The AUCs for our method and for the meta-analysis method drop at the same speed as the number of data sets increases, and our method results in higher AUCs in all scenarios. This is because both methods have to add noise to outputs from each private data set. Given a fixed number of observations (8,668), more noise is added when there are more private data sets (evenly split). The public data based method is not affected by this setting. In the experiments of this section, AUCs have small standard deviations (around 0.05) and the mean of results from 100 independent experiments is stable enough to represent performance of the methods (the standard deviation of the mean is around 0.005). Therefore, we only plot the mean AUCs).

Figure 1b shows the AUCs of the models given different fractions of public data. Our algorithm's performance is stable, but the AUCs of the public data based model grow quickly with increased sample size. When the fraction of public data is between 1% and 5%, our algorithm is the best.

Figure 1c shows how AUCs change at different regularization strengths ( λ in Algorithm 2). Our method has the most stable performance even when the regularization strength increases 100 times, or when it is 10% of the optimal value. This is very important in practice, as it is expensive to calculate the best regularization strength (i.e., have to reserve additional test data for tuning regularization strength, which will also spend some privacy budget). With the stable performance, we can hypothesize that even if the guess is away from the optimal regularization strength, our method can perform well in terms of discrimination.

Figure 1d shows the effect of the privacy budget ( in Algorithm 2) on AUCs. The method based on public data is not affected, but the other two have better performance with more budgets. Our algorithm is better than the meta analysis model in general, and outperforms the public data based method when the privacy budget is larger than 1.

Figure 1e shows how the number of iterations in distributed logistic regression algorithm ( l in Algorithm 2) will affect our algorithm's performance. When there are no iterations (i.e., only locally calculated parameters are used), our method degrades to the public data based algorithm (the same starting point). Our performance is best with less than 3 iterations, and it gets worse with more iterations. There are several reasons for the degraded performance. First, our algorithm uses parameters trained on public data set as the starting point, which is expected to be close to the real one, and therefore, the necessary iterations are smaller than the traditional Newton method (starting from all zeros). Second, as noise is added to each iteration, the gain from later iterations can be masked by the increased amount of noise. Yet another reason is that our privacy budget is evenly split into a pre-determined number of iterations, and more iterations imply a large amount of total noise.

A natural question is when our method would be most useful. This is a hard question to answer in theory because our method has no guarantee of convergence (due to the added noise). However, we can answer the question empirically.

We decompose parameters into "external factors" and "controllable factors". The former corresponds to: the number of private data sets, the fraction of data that are public, and the private budget (set by the data owner), which researchers cannot control. The regularization strength and iteration numbers, however, are not external factors, as researchers can choose them.

**Figure 2:**
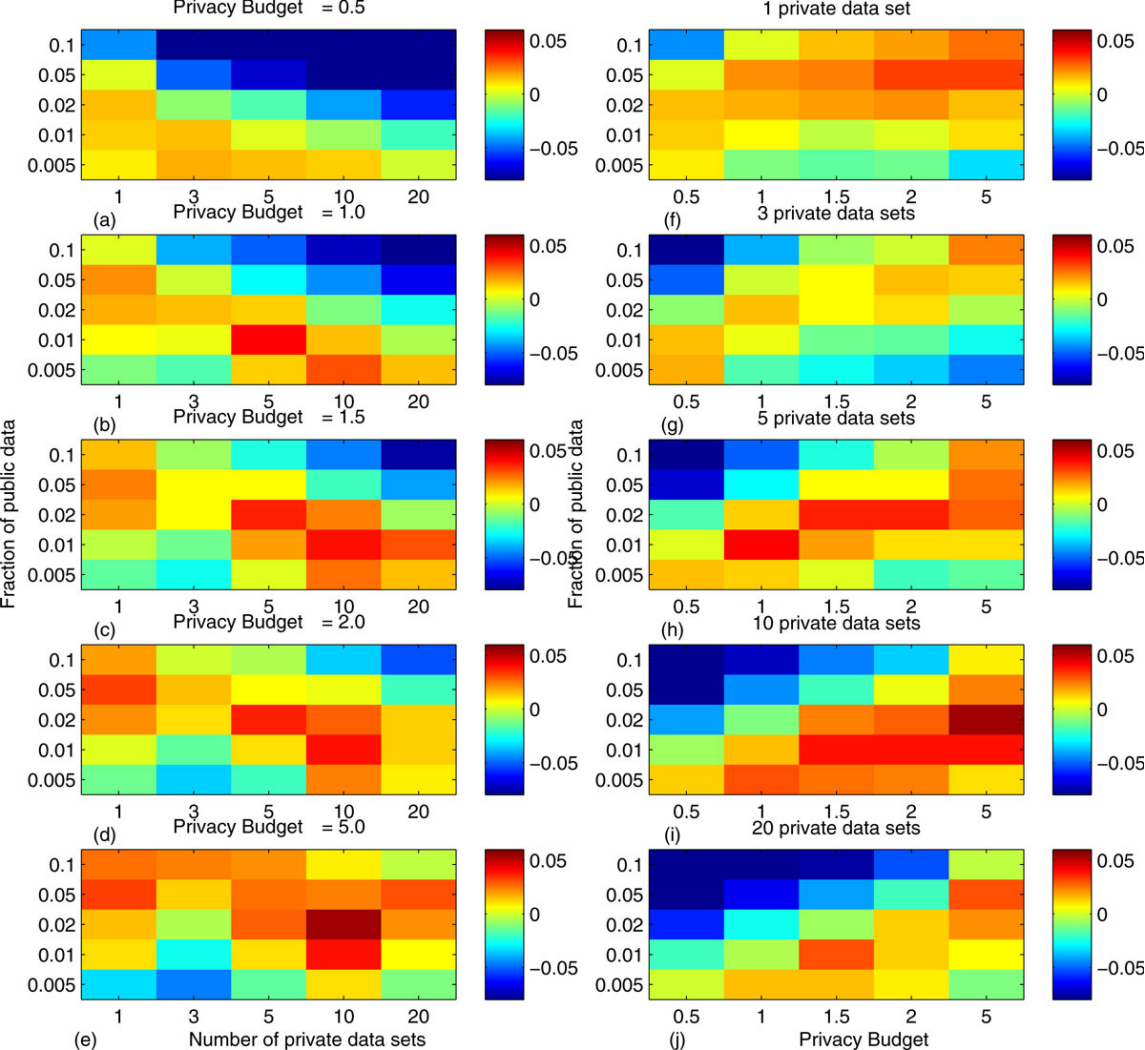
Comparison of three methods given different external settings: the number of private data sets, the fraction of public data, and the privacy budget. Red and yellow rectangles indicate our algorithm outperforms the other methods, while green and blue rectangles mean the opposite.

In Figure 2, we showed how the three methods perform (Using their corresponding "best" controllable factors) given different external factors, which include the numbers of private data sets (1, 3, 5, 10 and 20), the percentages of public data (0.5%, 1%, 2%, 5%, 10%) and the privacy budgets (0.5, 1, 1.5, 2, 5). Each rectangle represents a comparison between our method and the best of the other two approaches. Red and yellow rectangles indicate our algorithm is better, while green and blue rectangles mean the opposite. Our algorithm performs well under many situations, including: (1) privacy budget equal or larger than 1, (2) 5-10 private data sets, (3) percentage of public data around 1-5%.

*2) Model comparison using different datasets: *Our last study evaluated model performance using data of different sizes. Three biomedical datasets used in this experiment differ in the number of observations. All input parameters are set to default values. Each experiment is repeated 100 times to generate a boxplot.

**Figure 3:**
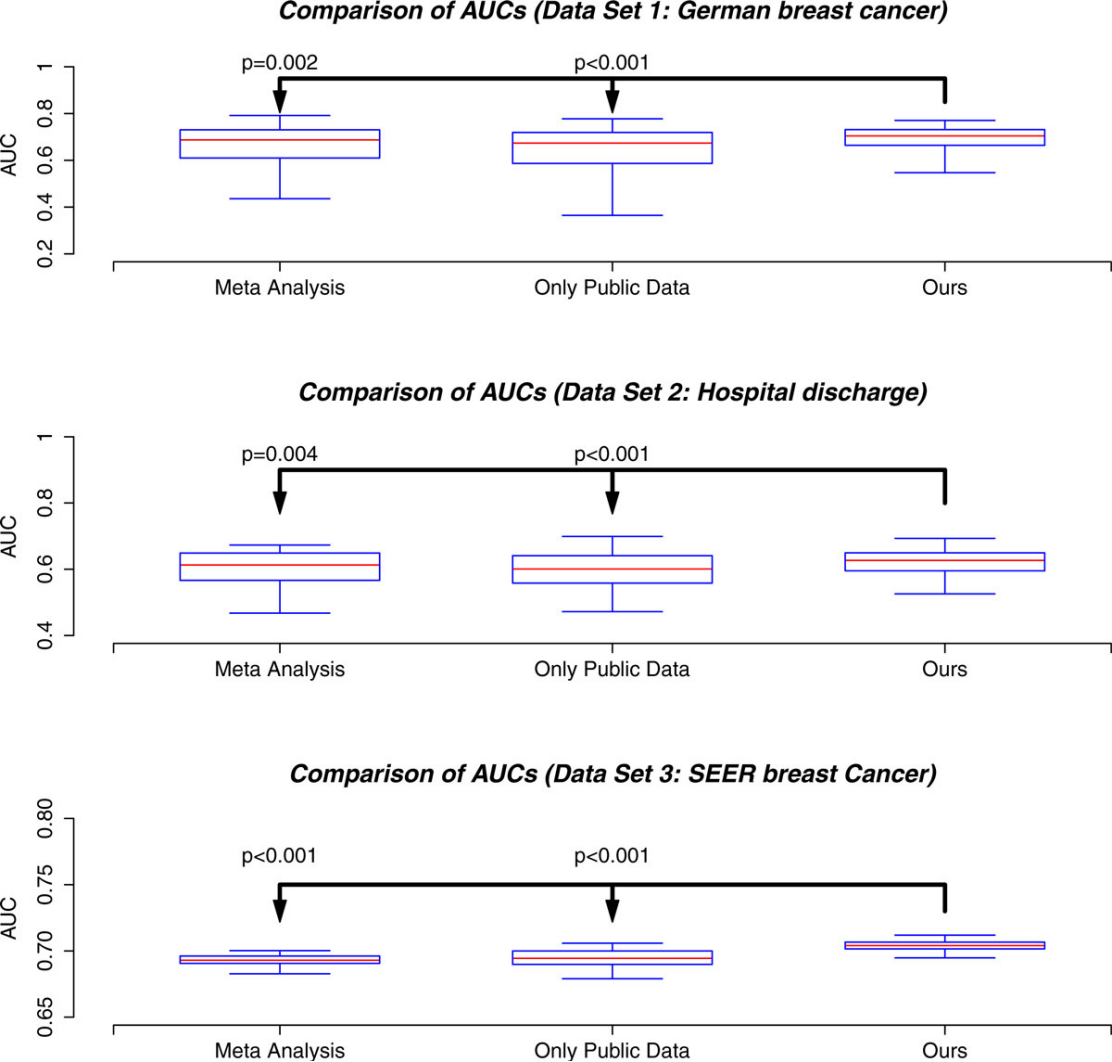
Boxplot comparisons of models using three different datasets. We use default parameter values as stated in the begining of this section. For each method, the five lines from bottom to top are 2.5%, 25%, 50%, 75% and 97.5% quantiles of AUCs. The p-value of pairwise t-test on AUCs are also shown.

In Figure 3, our algorithm is compared to the meta-analysis model and the logistic regression model trained on public data sets. In all the three data sets, our algorithm shows the best performance as indicated by the p-value (the p-values are calculated using the pairwise one-sided student-t test).

## Discussion and conclusion

We demonstrated a novel approach that combines public and private data sets to build a logistic regression model in a distributed manner. Our approach shows performance advantage over two other approaches under various conditions. There are still challenges in using our approach in practice. For example, categorical attribute values in private data sets may not appear in public data. The simplest solution is to pre-process private data sets by dropping values that rarely appear (or do not appear) in public data and that seem uncorrelated to the labels. This will not spend privacy budget as we only use public data to guide the process. It may improve a model's utility by removing some values and reducing the number of attributes. The upper bound of $L_{2}$ norm for the predictors therefore gets reduced, and so does the scale of noise.

Another limitation of our method is that we assume that all data sets follow the same (joint) distribution. However, it is possible that some data sets have sample bias, but may still follow the same learning rule. A solution for this is to use gradient descent instead of the Newton method in Algorithm 1. However, as gradient descent algorithm usually needs more iterations to get an accurate solution, and consequently it may add more noise and therefore generate less satisfactory outputs. In conclusion, we propose a new algorithm to extend the differential private framework to real world scenarios in biomedical research, where public and private data sets are available for analysis. Hybrid approaches that rigorously protect private data while leveraging public data to improve the utility show great promise to achieve "the best of both worlds" (i.e., data privacy and usefulness).

## Authors' contributions

ZJ contributed to the majority of the writing and experiments. XJ proposed the idea of learning a logistic regression with private and public datasets. He wrote introduction and contributed to the experiment design. SW and LX contributed to the algorithm development and helped with the experiments. LOM oversees this research project and provided thorough edits to the different versions of the manuscript.

## Competing interests

We have no conflict of interest.

## Supplementary Material

Additional file 1 Click here for file

## Declarations

Funding for the publication of this article has partly come from NIH grants R00LM011392, U54HL108460, UL1TR0001000, AHRQ grant R01HS019913 and PCORI contracts.

This article has been published as part of *BMC Medical Genomics *Volume 7 Supplement 1, 2014: Selected articles from the 3rd Translational Bioinformatics Conference (TBC/ISCB-Asia 2013). The full contents of the supplement are available online at http://www.biomedcentral.com/bmcmedgenomics/supplements/7/S1.
