# Supplementary material for: Differentially private distributed logistic regression using private and public data
Source: BMC Med Genomics. 2014 May 8;7(Suppl 1):S14. doi: 10.1186/1755-8794-7-S1-S14 (PMC4101668; doi:10.1186/1755-8794-7-S1-S14)
Supplement: Additional file 1 [file 1755-8794-7-S1-S14-S1.docx]

Appendix

1. *Further Improvements*

To further improve performance, we propose some additional modifications to our model. Because the size of the public data set is much smaller than the size of the private data sets, the sample error of the public data set is much larger. Therefore, it is better to enforce a larger regularization and replace the coefficient $\frac{n_{0}\lambda}{\sum_{j=0}^{k} n_{j}}$ in the Hessian matrix (Step 1 in Algorithm 1) with $\lambda$.

Another improvement to our plain model (which starts with all zero $\beta$) is to initiate $\beta$ parameters with the ones trained on the public data set. Although the parameters learned from the small public data set are influenced by the large sampling error, they are still closer to the true parameters than zeros. With a better prior, we can spend privacy budgets more effectively.

1. *Proof of Differential Privacy*

For each private data set, the only outputs are the gradients in each of the *l* iterations. Since the privacy budget in each step is additive (i.e., sequential composition property), we only need to prove that each iteration takes $\epsilon_{0}=\epsilon/l$ privacy budget. Using the Laplacian mechanism (Definition 4), we only need to prove that the $L_{2}$ sensitivity for the log-likelihood’s gradient in each data set is $2M$.

Because the gradient of the log-likelihood function on the $j$-th private data set is $\sum_{i=1}^{n_{j}} \frac{y_{i}^{j}x_{i}^{j}}{1+\exp(y_{i}^{j}\beta_{old}^{T}x_{i}^{j})}$, the change of gradient when sample $(x^{*},y^{*})$ is replaced by $\left( x^{**},y^{**} \right)$ is

$$\frac{x^{**}y^{**}}{1+\exp\left( y^{**}\beta^{T}x^{**} \right)}-\frac{x^{*}y^{*}}{1+\exp\left( y^{*}\beta^{T}x^{*} \right)}$$

As $\frac{x^{**}y^{**}}{1+\exp\left( y^{**}\beta^{T}x^{**} \right)}$ and $\frac{x^{*}y^{*}}{1+\exp\left( y^{*}\beta^{T}x^{*} \right)}$ are both scalars, whose absolute values are no more than 1, the norm of this change is less than the sum of $x^{**}$’s norm and $x^{*}$’s norm. Both $x^{**}$’s and $x^{*}$’s norms are less than $M$ (Algorithm 1), therefore, the sensitivity of gradient in each data set is $2M$. According to the Laplacian mechanism, Algorithm 1 is $\epsilon_{0}$ differentially private, and according to the sequential composition property of differential privacy, Algorithm 2 is $\epsilon$ differentially private.
